# In‐the‐Clinic series: A 10 year journey

**DOI:** 10.1002/btm2.70137

**Published:** 2026-03-16

**Authors:** Samir Mitragotri

**Affiliations:** ^1^ School of Engineering & Applied Sciences Harvard University Allston Massachusetts USA; ^2^ Wyss Institute for Biologically Inspired Engineering at Harvard University Boston Massachusetts USA

*Bioengineering & Translational Medicine* was launched in 2016 with the goal of publishing manuscripts relevant to researchers in biomedicine and translational science, particularly within the AIChE community. To advance this mission, the journal introduced a series of In‐the‐Clinic articles to provide readers with concise summaries of the clinical landscape across various fields.

The purpose of launching this series was straightforward: to offer practical guidance to researchers engaged in translational medicine. A critical first step in developing a new biomedical technology is gaining a comprehensive understanding of existing solutions available to patients, as well as those advancing through clinical trials. The development of a new clinical technology is a resource‐ and time‐intensive endeavor. Therefore, it is especially important for researchers to understand which solutions are ahead in the developmental timeline, as such insight can meaningfully inform unmet need, indication selection, and differentiation strategy.

Over the past 10 years, we have published In‐the‐Clinic articles spanning a wide range of technologies, including nanoparticles, hydrogels, viral vector‐based gene therapies, hemostats, RNA therapeutics, PEGylated therapeutics, digital therapeutics, cell therapies, cancer vaccines, therapies for Alzheimer's and Parkinson's diseases, antibody‐drug conjugates, stem cell therapies, and vaccine adjuvants for infectious diseases. Collectively, these articles have analyzed over 10,000 Phase I, II, and III clinical trials and provided multidimensional summaries of the clinical landscape. As the clinical landscape evolves, we have updated these articles every 2–3 years to ensure they contain current and relevant information.

This series has been exceptionally well received, as reflected by approximately 300,000 downloads worldwide over the years. Overall, *Bioengineering & Translational Medicine* articles have been downloaded in more than 200 countries and territories, and the In‐the‐Clinic series has contributed significantly to the journal's strong global reach.

Beyond serving as a valuable resource for readers, the series has also provided an important learning experience for its authors. Contributors have included high school, undergraduate, and graduate students, as well as postdoctoral researchers. Participation in writing these reviews has offered trainees a unique opportunity to develop a deep understanding of the clinical landscape. I am deeply grateful to all co‐authors who have made these articles possible. I am especially thankful to Dr. Aaron Anselmo, who championed the planning and writing of this series during the launch of the journal, and to Prof. Zongmin Zhao, who has carried forward this responsibility in recent years. Special thanks also to Prof. Elizabeth Nance, Editor in Chief, for her support of this series and its publication in the journal.

It has been my pleasure and honor to lead the In‐the‐Clinic series over the past decade. I hope that readers of the journal have found these articles valuable and that they have meaningfully helped guide their own translational endeavors.

## AUTHOR CONTRIBUTIONS

**Samir Mitragotri:** Conceptualization; writing – original draft; writing – review and editing.

## CONFLICT OF INTEREST STATEMENT

The author declares no conflicts of interest.

